# Antibody-drug conjugates in urinary tumors: clinical application, challenge, and perspectives

**DOI:** 10.3389/fonc.2023.1259784

**Published:** 2023-12-20

**Authors:** Keqiang Li, Guoqing Xie, Xiyue Deng, Yu Zhang, Zhankui Jia, Zhenlin Huang

**Affiliations:** ^1^ Department of Urology, the First Affiliated Hospital of Zhengzhou University, Zhengzhou, Henan, China; ^2^ Academy of Medical Sciences, Zhengzhou University, Zhengzhou, Henan, China

**Keywords:** ADC, urinary tumors, kidney, bladder, urothelial cancer, prostate

## Abstract

Urinary tumors primarily consist of kidney, urothelial, and prostate malignancies, which pose significant treatment challenges, particularly in advanced stages. Antibody-drug conjugates (ADCs) have emerged as a promising therapeutic approach, combining monoclonal antibody specificity with cytotoxic chemotherapeutic payloads. This review highlights recent advancements, opportunities, and challenges in ADC application for urinary tumors. We discuss the FDA-approved ADCs and other novel ADCs under investigation, emphasizing their potential to improve patient outcomes. Furthermore, we explore strategies to address challenges, such as toxicity management, predictive biomarker identification, and resistance mechanisms. Additionally, we examine the integration of ADCs with other treatment modalities, including immune checkpoint inhibitors, targeted therapies, and radiation therapy. By addressing these challenges and exploring innovative approaches, the development of ADCs may significantly enhance therapeutic options and outcomes for patients with advanced urinary tumor.

## 1 Introduction

Urinary malignancies, primarily encompassing kidney, urothelial, and prostate cancers, pose a significant global health buren. Prostate cancer is the most frequently diagnosed cancer in men, whereas bladder cancer holds the tenth position (1–5). The diverse nature of these cancers necessitates a range of treatment strategies, tailored according to the stage and characteristics of the disease. Therapeutic approaches span from surgical interventions, radiotherapy, chemotherapy, to targeted and immune therapies, each with its unique limitations.

Renal cell carcinoma (RCC), most common type of kidney cancer, presents a diverse clinical landscape with varying outcomes. Surgery, specifically nephrectomy, is typically the standard treatment. However, this approach may result in postoperative complications and impaired renal function. For metastatic RCC, targeted therapies, such as inhibitors of VEGF/VEGFR and mTOR, have demonstrated potential, but resistance often develops within 6 to 15 months, undermining their long-term effectiveness. Immunotherapy, despite its potential, has a relatively low response rate and frequently presents substantial toxicity (6, 7).

In addition, urothelial cancer, a malignancy occurring in the bladder, ureters, and renal pelvis, is also primarily managed with surgical intervention. However, recurrence following transurethral resection or radical surgery is a significant concern. For advanced or metastatic urothelial cancer, platinum-based chemotherapy is the first-line treatment. Even though response rates can reach up to 60%, it’s far from the ideal. The second-line treatment typically involves immunotherapies, such as inhibitors of PD-1/PD-L1, but these often yield disappointingly low response rates and immune-related adverse effects (5, 8, 9).

Meanwhile, prostate cancer is primarily managed with radical prostatectomy for localised disease. Yet, the procedure carries risks such as urinary incontinence and erectile dysfunction. Radiotherapy provides an alternative for localised prostate cancer, but it has its own drawbacks, including rectal and genitourinary toxicity. For metastatic prostate cancer, androgen deprivation therapy is fundamental, but the eventual onset of castration resistance limits its long-term effectiveness (10–13).

Despite advancements in various therapeutic approaches, the prognosis for advanced and metastatic urinary tumor remains dismal due to limited treatment options and the development of resistance mechanisms. Traditional chemotherapy is often associated with significant side effects and may not be suitable for all patients.

In recent years, antibody-drug conjugates (ADCs) have emerged as a promising therapeutic approach for urinary tumors. ADCs deliver a targeted cytotoxic payload to cancer cells while minimising off-target effects, potentially providing a safer alternative for patients with compromised renal function. However, challenges persist in the development and optimisation of ADCs for urinary tumor treatment, including managing toxicities, identifying predictive biomarkers, and overcoming resistance. Further investigations into combining ADCs with other treatments could potentially enhance efficacy and overcome resistance (14, 15).

In this review, we will examine the current landscape of ADCs in urinary tumor treatment, highlighting recent advancements, opportunities, and challenges in the field. Our focus will be on the application of ADCs in kidney, urothelial, and prostate cancer, and we will scrutinise the potential of novel ADC targets and combination therapies.

## 2 About antibody-drug conjugates (ADCs)

Antibody-drug conjugates (ADCs) represent a promising class of targeted cancer therapeutics that have their origins in Paul Ehrlich’s vision of “magic bullets” in 1913 (16). This concept has since evolved, with the first ADCs making use of mouse antibodies entering clinical trials in the 1980s (17, 18). The field has witnessed significant progress over the years, leading to the FDA approval of 15 ADCs: Gemtuzumab ozogamicin (Mylotarg) in 2000, Brentuximab vedotin (Adcetris) in 2011, Trastuzumab emtansine (Kadcyla) in 2013, Inotuzumab ozogamicin (Besponsa) in 2017, Moxetumomab pasudotox (Lumoxiti) in 2018, Polatuzumab vedotin (Polivy) in 2019, Enfortumab vedotin (Padcev) in 2019, Trastuzumab deruxtecan (Enhertu) in 2019, Sacituzumab govitecan (Trodelvy) in 2020, Cetuximab saratolacan (Akalux) in 2020, Belantamab mafodotin (Blenrep) in August 2020 (then withdrawn), Loncastuximab tesirine (Zynlonta) in 2021, Tisotumab vedotin (Tivdak) in 2021, Disitamab Vedotin (Aidixi) in 2021, and Mirvetuximab soravtansine (Elahere) in 2022. It is noteworthy that most of these 15 ADCs have utilised the FDA’s accelerated approval process, reflecting both the need for further confirmation of their ultimate efficacy and the immense potential of ADCs in treating end-stage cancer (19, 20) (**Table 1**).

**Table 1:** Antibody-Drug Conjugates approved by the FDA.

ADC	Target Antigen	Payload	Payload Target	Indication	Approval
Gemtuzumab ozogamicin (Mylotarg)	CD33	Calicheamicin	DNA	CD33+ acute myeloid leukemia	2000 (withdrawn 2010) re 2017
Brentuximab vedotin (Adcetris)	CD30	MMAE	Tubulin	Relapsed or refractory and previously untreated stage III/IV Hodgkin lymphoma	2011
				Relapsed or refractory and untreated systemic anaplastic large cell lymphoma	
Trastuzumab emtansine (Kadcyla)	HER2	DM1	Tubulin	HER2+ metastatic breast cancer	2013
Inotuzumab ozogamicin (Besponsa)	CD22	Calicheamicin	DNA	Relapsed or refractory B-cell acute lymphoblastic lymphoma	2017
Moxetumomab pasudotox (Lumoxiti)	CD22	Pseudomonas exotoxin	eEF-2	Relapsed or refractory hairy cell leukemia	2018
Polatuzumab vedotin (Polivy)	CD79b	MMAE	Tubulin	Diffuse large B-cell lymphoma	2019
Enfortumab vedotin (Padcev)	Nectin-4	MMAE	Tubulin	**Advanced or metastatic urothelial cancer**	2019
Trastuzumab deruxtecan (Enhertu)	HER2	DXd	DNA (topoisomerase)	Unresectable or metastatic HER2+ and HER2- breast cancer	2019
				Unresectable/metastatic HER2+ non-small cell lung cancer	
Sacituzumab govitecan (Trodelvy)	Trop-2	SN-38	DNA (topoisomerase)	Unresectable or metastatic triple negative breast cancer **Advanced or metastatic urothelial cancer**	2020
Belantamab mafodotin (Blenrep)	BCMA	MMAF	Tubulin	Relapsed or refractory multiple myeloma	2020 (withdrawn 2022)
Cetuximab saratolacan (Akalux)	EGFR	IRDye700DX	Cell membrane	Recurrent head and neck cancer	2020
Loncastuximab tesirine (Zynlonta)	CD19	SG3199/PBD dimer	DNA	Relapsed or refractory large B-cell lymphoma, diffuse large B-cell lymphoma	2021
Tisotumab vedotin (Tivdak)	Tissue factor	MMAE	Tubulin	Recurrent or metastatic cervical cancer	2021
Disitamab Vedotin (Aidixi)	HER2	MMAE	Tubulin	HER2+ advanced or metastatic gastric cancer (including gastroesophageal junction adenocarcinoma)	2021
				**HER2+ advanced or metastatic urothelial cancer**	
Mirvetuximab soravtansine (Elahere)	FRα	DM4	Tubulin	FRα+ platinum-resistant epithelial ovarian, fallopian tube, or primary peritoneal cancer	2022

Bold words mean Urinary Tumours.

Two ADCs in particular warrant discussion: Belantamab mafodotin and Gemtuzumab ozogamicin. Belantamab mafodotin received accelerated FDA approval in August 2020 as a monotherapy for adult patients with relapsed or refractory multiple myeloma (MM). However, this approval was later withdrawn when the drug failed to achieve the expected results in subsequent phase III clinical trials (21). The story of Gemtuzumab ozogamicin is even more complex. As the first ADC approved by the FDA, it was withdrawn from the market in 2010 due to concerns about drug toxicity. After adjusting the dosage and conducting numerous clinical trials, Gemtuzumab ozogamicin was reapproved by the FDA in 2017 for use in newly diagnosed and relapsed/refractory CD33-positive acute myeloid leukemia patients (22, 23).

The withdrawal of these ADCs serves as a constant reminder that the risks associated with ADCs still require further evaluation. In order to mitigate the risks of ADCs, researchers should continue to actively explore new ADC manufacturing processes to achieve higher safety profiles. Furthermore, lower drug prices are needed to obtain larger-scale clinical data on treatment outcomes, as the amount of clinical data available for ADCs is still relatively limited when compared to traditional systemic chemotherapy. This will ultimately help to strengthen the logical flow and coherence of the information presented, contributing to a more robust understanding of ADCs and their potential in cancer treatment.

### 2.1 The three key components of ADC and development strategies

**Figure 1:**
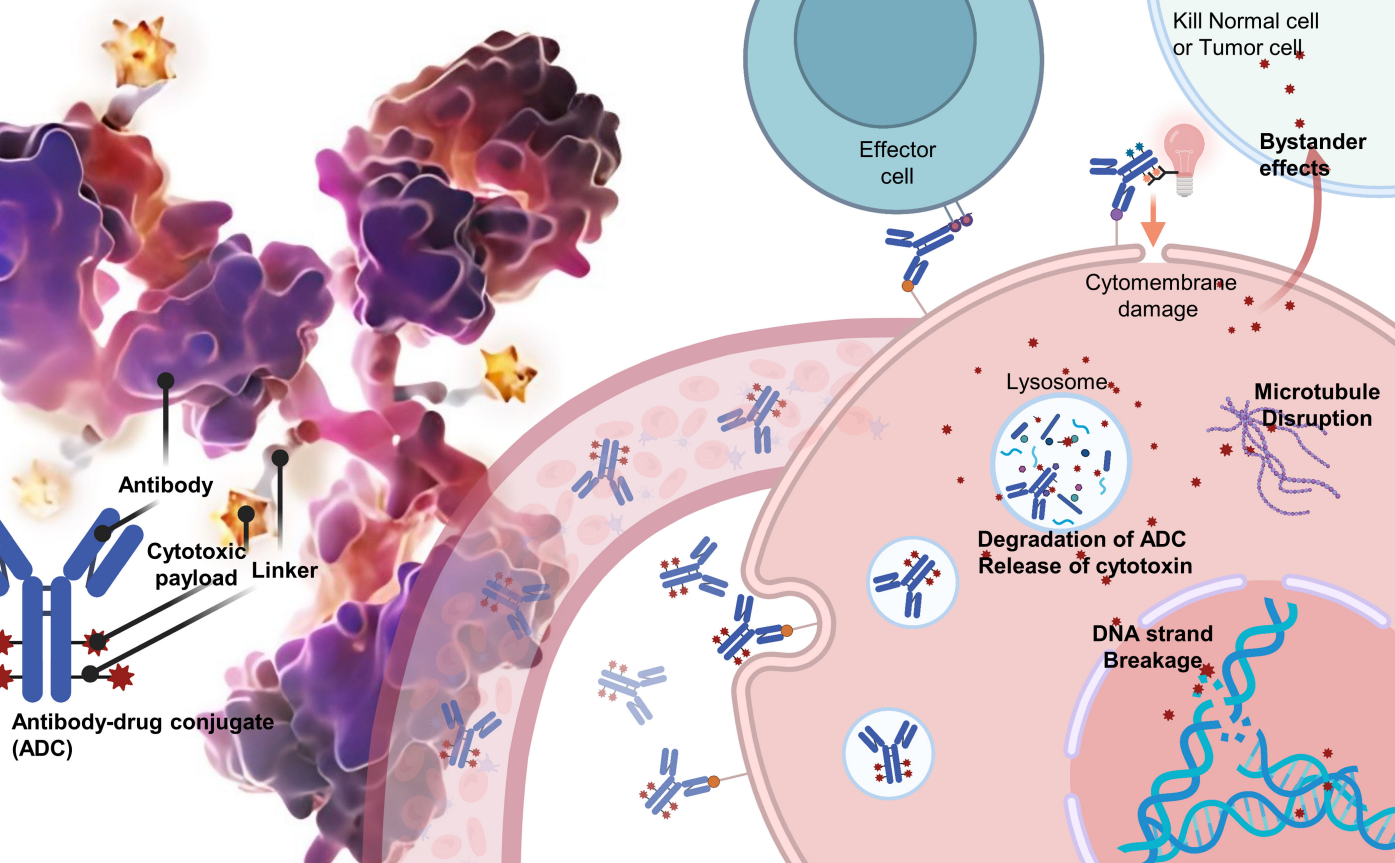
The core components and mechanism of antibody–drug conjugates (ADCs).

ADCs are composed of three integral components: a monoclonal antibody that selectively targets tumor antigens, a cytotoxic payload designed to disrupt DNA or tubulin within the targeted cancer cells, and a linker that covalently attaches the antibody to the payload. The efficacy of ADCs depends on the specificity and affinity of the antibody, the potency of the payload, and the stability of the linker in circulation, as well as its rapid release within the targeted cells (**Figure 1**).

Over the years, various conjugation modalities and linker chemistries have been investigated to optimise ADC development. Early ADCs primarily relied on lysine-based or cysteine-based chemical conjugation methods, which resulted in heterogeneous products with variable drug-antibody ratios (DARs) (24, 25). This heterogeneity often negatively impacted ADC efficacy and therapeutic windows, leading to failures in clinical trials. To overcome these issues, researchers have turned to alternative strategies, such as engineered cysteines, non-natural amino acids, and enzymatic conjugation using sortase or transglutaminase (26–28). These techniques enable the production of homogeneous ADCs with controlled DARs that exhibit improved pharmacokinetics and clinical outcomes.

Linker properties play a vital role in ADC stability, cytotoxicity, and pharmacokinetics. Traditionally, cleavable linkers have been used, which are designed to release payloads intracellularly in response to pH changes or the action of specific enzymes. In contrast, non-cleavable linkers necessitate complete antibody degradation for payload release. Current research is focused on developing novel linkers that offer optimal stability, solubility, and release profiles, as well as site-specific conjugation methods (24, 25).

In addition to linker design, the selection of appropriate target antigens and payloads is crucial to the potency of ADCs. An ideal target antigen is highly and uniformly expressed on tumor cells, but minimally expressed on healthy cells. However, antigen density does not always correlate well with ADC potency due to differences in internalisation rates. Therefore, numerous candidate target antigens are currently under investigation. The First-Generation ADCs payload ideally should have picomolar potential against tumor cells while demonstrating low off-target toxicity. Thus, the majority of ADC payloads have been DNA-damaging agents aimed at DNA and anti-mitotic agents targeted at tubulin, due to their differential toxicity to cancer and non-cancer cells (19, 29, 30). To overcome the remaining high toxicity limits of existing ADCs, researchers are using payloads with lower off-target toxicity and high DARs, while also increasing the solubility of the payloads to minimise damage to normal cells and achieve a larger therapeutic index and improved pharmacokinetics (29, 31, 32). Additionally, innovative attempts have been made in ADCs payload, such as the FDA-approved Cetuximab saratolacan (Akalux) in 2021, which uses the near-infrared photoimmunotherapy (NIR-PIT) agent IRdye700DX as a payload to reduce ADC toxicity to normal tissues. With these novel payloads, it is possible to overcome drug resistance mechanisms and achieve higher potency, ultimately leading to enhanced efficacy of ADCs and improved clinical outcomes (33, 34) (**Supplementary Material 1**).

### 2.2 Challenges and prospects for ADCs

Promising advances in the field of ADCs include engineered linkers and site-specific conjugation methods for improved homogeneity and stability, novel payload mechanisms to overcome resistance, alternative protein scaffolds for enhanced tumor penetration, and combination therapies with immune checkpoint inhibitors. A key aspect of these innovations is the introduction of high DARs, which enables targeting of membrane proteins with lower levels of expression than before. This breakthrough has made it possible for ADCs to be designed on highly specific, yet less abundant targets. Moreover, it also allows for a reduction in the dosage of ADCs administered to conventional targets, thereby enhancing the potential and efficacy of ADCs. Consequently, these innovations have facilitated the development of highly optimised ADCs with improved pharmacokinetics and clinical outcomes (35).

The ADC landscape has experienced significant growth in recent years, with over 60 ADCs currently in clinical trials. Continued advancements in medicinal chemistry and pharmacology will undoubtedly foster innovative cancer therapeutics and bring us closer to realizing personalized ADC-based cancer treatments.

Despite these promising developments, challenges remain in selecting optimal target antigens, identifying highly potent and selective payloads, and devising robust manufacturing processes for the large-scale production of ADCs. Moreover, further insights are needed regarding ADC biotransformations, toxicity profiles, and resistance mechanisms (35).

To encapsulate, ADC technology represents an emerging molecular platform that is expected to become a mainstay in anti-cancer therapeutics. While the target antigens and payloads are crucial, it is equally important to develop optimal antibody-payload conjugation methods and linker chemistries. Further investigations from medicinal chemistry and pharmacology standpoints into linker/conjugation strategies, payloads, and protein scaffolds will likely lead to innovative cancer therapeutics in the near future.

ADCs, as a significant achievement in precision medicine, have introduced new strategies for cancer treatment. With the ongoing advancements in ADC technology, coupled with a deeper understanding of the unique challenges posed by urologic cancers, we are undoubtedly on the brink of more effective and personalised treatment approaches for patients afflicted with these devastating diseases.

## 3 ADCs in urinary tumor

### 3.1 Kidney cancer and ADCs

#### 3.1.1 Overview of kidney cancer and current treatment landscape

Kidney cancer, particularly renal cell carcinoma (RCC), is primarily treated with surgery. However, for advanced and metastatic kidney cancer, the treatment goal is mainly to control disease progression, prolong patient survival, and improve quality of life. Regrettably, RCC is not sensitive to radiotherapy and chemotherapy and is prone to resistance. Therefore, over the past 20 years, treatment of RCC has evolved from immune cytokines (such as IL-2 and IFN-α) to tyrosine kinase inhibitors (TKI) targeting vascular endothelial growth factor receptor (VEGF), mTOR, or ALK. The use of immune checkpoint inhibitors has resulted in breakthrough progress in RCC treatment. However, overall, non-surgical treatment options for RCC are less effective compared to other urological cancers (36–38).

#### 3.1.2 Advancements and opportunities in kidney cancer ADCs

ADCs are a class of novel targeted drugs and are emerging as potential treatment options for kidney cancer. Compared to traditional non-surgical options, due to the targeted characteristics and high toxicity of ADCs, they can significantly mitigate the damage to normal cells while reducing the likelihood of RCC resistance. Given that renal function in kidney tumor patients is further impaired compared to other cancers, and metabolic capabilities are weakened, combined with the high resistance potential of RCC, ADCs targeting kidney cancer have broad application prospects.

Currently, progress in ADCs for kidney cancer mainly focuses on two aspects. Firstly, the technical iteration of ADCs has made site-specific conjugation of payloads possible. Enhanced by third-generation ADC technology, ADCs characterized by high DARs and low off-target toxicity present a more suitable therapeutic strategy for renal tumors, which are particularly challenging due to their lack of stable targets and heightened sensitivity to toxicity (35). This might bring some less-than-ideal targets back into consideration, such as CD70, which has been the focus of multiple clinical trials for kidney cancer ADCs, but has not produced satisfactory results (39). Secondly, finding new renal tumor-specific targets has always been a crucial breakthrough direction for kidney cancer ADCs application, such as CD56, CDCP1, and CDH6 (40–42). Moreover, although ENPP3 and TIM-1 have received less attention in research, their high expression in kidney cancer and low expression in normal tissues make them potential targets for antibody-drug conjugates, warranting further study (43, 44).

#### 3.1.3 Challenges and future directions for kidney cancer ADCs

Currently, no ADC has been approved for RCC, nor are any ADCs being evaluated in phase II/III trials (45). This represents an opportunity but also indicates significant challenges in designing ADCs for kidney cancer, distinct from other urological cancers. The main issue is that renal tumors lack specific and less heterogeneous stable targets, leading to poor initial clinical trial performance of most kidney cancer ADCs, even those that are effective in other cancers.

Compared to other urological cancers, the application of ADCs with nephrotoxic payloads in renal tumors need exceptional caution (e.g., a-amanitin). Even many non-nephrotoxic payloads need to reduce doses considering the severe renal damage and insufficient metabolic capabilities in kidney cancer patients. However, the reduced dosage and toxicity of ADCs could, in turn, promote RCC resistance, especially given the renowned resistance of kidney cancer. These factors make the development of ADCs for kidney cancer challenging.

Despite these obstacles, researchers haven’t abandoned this promising realm. Besides optimising ADC production processes and seeking specific targets for renal tumors, considering the high vascularisation characteristic of renal tumors, the combined use of ADCs with drugs targeting VEGF, such as TKIs, might produce good results (38, 46). Anti-angiogenesis drugs can increase “pressure” on the tumor, subsequently enhancing the expression of specific antigens on the tumor cell surface, thereby enhancing the effect of ADCs, much like the combination of TKIs and immune checkpoint inhibitors (47).

### 3.2 Urothelial cancer and ADCs

#### 3.2.1 Overview of urothelial cancer and current treatment landscape

Urothelial carcinoma, primarily bladder cancer, progresses slowly but tends to recurrence. This is closely related to the physiological structure of the urinary tract. On one hand, most urothelial carcinomas only grow within the epithelial tissue of the urinary tract and do not penetrate the muscle layer. On the other hand, while most urothelial carcinomas can be directly inspected and treated through the urethra in an intuitive and less damaging way, this approach is also likely to leave remnants. However, once non-invasive urothelial carcinoma develops into invasive or metastatic urothelial carcinoma, the situation becomes challenging. Generally, there are various treatment modalities for urothelial carcinoma, with surgery, radiotherapy, and chemotherapy displaying effective outcomes. Even intravesical instillation chemotherapy serves as a first-line treatment plan in bladder cancer. It’s worth noting that not all instilled drugs are chemotherapeutics. BCG and Pseudomonas aeruginosa work by altering the tumor microenvironment, promoting immune cell aggregation, and exerting anti-cancer effects (5, 48, 49).

#### 3.2.2 Advancements and opportunities in urothelial cancer ADCs

Research on ADCs for urothelial carcinoma is the most mature in urological tumors, notably illustrated by the FDA’s approval of enfortumab vedotin and sacituzumab govitecan for use in late-stage and previously treated patients. Compared to traditional chemotherapy regimens (such as paclitaxel, docetaxel, or vincristine), enfortumab vedotin significantly improves overall survival (50–53).

The extensive research into the application of Antibody-Drug Conjugates (ADCs) in urothelial carcinoma, coupled with promising clinical trial results, is underpinned by the presence of more tumor-specific and abundant antigens inherent to this form of cancer. The targets of ADCs for urothelial carcinoma are mainly the three membrane proteins: HER-2, TROP2, and Nectin-4 (54–56). Interestingly, among the three membrane proteins, researchers and manufacturers seem to favour the HER-2 target. This is partly because HER-2 is overexpressed in various tumors, hence the designed ADCs may be applicable to multiple tumors. For instance, Disitamab Vedotin (Aidixi) can be applied in both urothelial carcinoma and gastric cancer (56). On the other hand, it’s worth exploring whether ADCs originally designed for other tumors with HER-2 targets will also work in urothelial carcinoma, such as Trastuzumab emtansine (Kadcyla), which the FDA approved for use in breast cancer in 2013 (57). Subsequently, researchers started investigating whether this ADC could also be used in urothelial carcinoma which also overexpresses HER-2 (58). Importantly, other ADCs targeting SLITRK6 and EpCAM are also under research, potentially greatly improving patient prognosis (59–61).

#### 3.2.3 Challenges and future directions for ADCs in urothelial cancer

Unlike the situation with renal cancer, where the utilization of ADCs is still in the primary stage of identifying appropriate targets, the challenges faced by urothelial carcinoma have advanced past this phase. The focus is gradually shifting towards achieving more universal applicability of ADCs in the treatment of urothelial carcinoma. This entails broadening the scope of usage in urothelial carcinoma and striking a new balance between improved prognosis and cost-effectiveness. ADCs primarily target advanced and metastatic tumors, but in the context of some early-stage urothelial carcinoma also utilise chemotherapy as a first-line treatment, it is worth exploring whether ADCs can improve patient prognosis in early-stage urothelial carcinoma. Notably, clinical trials have already begun to investigate the use of ADCs in early-stage urothelial carcinoma (NCT05014139) (62).

Due to the unique physiological structure of urothelial carcinoma, which allows for examination and even treatment via the urethra, intravesical instillation therapy has become a distinctive treatment method. Since the drug directly contacts the tumor and seldom enters the circulatory system, its efficacy and side effects are significantly less than those of traditional chemotherapy. If ADCs were applied in bladder intravesical instillation chemotherapy, considering the targeted nature of ADCs leading to fewer side effects and better efficacy, it could be possible to achieve better results and reduce patient discomfort with longer single-use instillation of ADCs. At present, very few studies on ADCs intravesical instillation therapy exist, with only a handful of animal simulation experiments conducted under experimental conditions. The main barrier to using ADCs in intravesical instillation is the high cost, especially considering the relatively low cost and side effects of traditional instillation chemotherapy, making the use of expensive ADCs seem unnecessary. In addition to the cost, there are also concerns about damage to normal bladder cells from the direct release of toxic payloads into the instillation fluid after the linker breaks when ADCs reaches the target. This is further complicated by the poor penetration abilities of the drugs used in instillation chemotherapy. Encouragingly, current animal experiment data indicate that the toxicity of the payloads does not cause more significant damage, and the overall effect of ADCs instillation is good (63, 64). Therefore, for urothelial carcinoma, considering the good targeting of ADCs, it is worthwhile to reduce costs, even at the expense of some efficacy, to achieve more widespread and universal application, thereby achieving collective benefits. Furthermore, the further development of ADCs in urothelial carcinoma could be driven by the large-scale clinical application data from universally applicable ADCs.

### 3.3 Prostate cancer and ADCs

#### 3.3.1 Overview of prostate cancer and current treatment landscape

Prostate cancer is the second most common cancer among men worldwide. However, due to the slow and confined growth of prostate tumors, the prognosis is generally favourable (10). Radical prostatectomy is the preferred treatment for prostate tumors, but for metastatic prostate cancer, postoperative radiotherapy and endocrine therapy are often necessary (11). Prostate tumors are not sensitive to chemotherapy, a characteristic that is closely related to their slow growth and the activation of androgen receptor (65–67). In recent years, we have witnessed the success of novel hormone therapies and PARP inhibitors as maintenance treatments following radical prostatectomy for prostate cancer, and the failure of immune checkpoint inhibitors in curing this malignancy. In summary, as the second most common tumor in men and with a long non-surgical treatment duration, a considerable amount of research is being poured into this field to find new treatments for early and late-stage prostate cancer.

#### 3.3.2 Advancements and opportunities in prostate cancer ADCs

Research and application of ADCs in prostate cancer have shifted from traditional targets (such as HER-2, TROP-2) to PSMA, a target with higher specificity in prostate tumors. PSMA is a protein that is highly expressed on the surface of prostate cells (68–70). Although PSMA can also be found in normal prostate cells, its expression usually significantly increases in prostate cancer cells. Additionally, the level of PSMA expression is associated with the severity of prostate cancer; higher-grade or more invasive cancers usually express more PSMA, which has greatly improved the efficacy of ADCs in the prostate (71, 72). Apart from PSMA, other prostate-specific targets are also under investigation, such as STEAP-1, B7-H3 and SLC44A4 (73–75).

Another advantage of using ADCs in prostate cancer is its close correlation with age (10). Therefore, for the older population prone to prostate cancer, the tolerance to the damage from chemotherapy becomes a major issue. The characteristic of ADCs having fewer side effects compared to traditional chemotherapy drugs give them a unique advantage when used in the elderly population.

#### 3.3.3 Challenges and future directions for prostate cancer ADCs

The slow growth of prostate tumors is a double-edged sword. On the one hand, patients are given a longer treatment window and a better prognosis. On the other hand, most chemotherapy drugs, including the toxic payloads in ADCs, function by inhibiting tumor cell division, which is inherently less effective for slow-growing prostate tumors. This is why the current application of ADCs in prostate tumors is not as effective as in urothelial carcinoma.

In addition, from the research on ADCs in prostate cancer, prostate tumors are not lacking targets with sufficient abundance and specificity (such as PSMA). Therefore, to achieve better therapeutic effects in prostate cancer, ADCs need innovative payloads, such as non-proliferative payloads. However, the definition of the payloads in ADCs does not stipulate that these payloads must be cytotoxic. Therefore, payloads that operate by recruiting effector cells, or through other mechanisms, may be worth further research.

## 4 Discussion

The advent of antibody-drug conjugates has marked a significant breakthrough in tumor therapy in recent years, particularly for various cancer types such as gastric, lung, and urinary tumors, where chemotherapy is the primary treatment. ADCs offer the advantage of increasing drug concentration at the tumor site to achieve superior anti-tumor effects with reduced drug dosage. This shift in therapeutic strategy has prompted researchers and clinicians to revaluate cancer treatment approaches.

Despite the encouraging clinical outcomes of ADCs in certain tumor types, numerous challenges persist. Firstly, the lack of suitable targets for some tumors, including kidney cancer, limits the application of ADCs. Secondly, ADCs are associated with severe complications such as bone marrow suppression, nausea, vomiting, dizziness, dyspnoea, and fatigue, some of which can be fatal. Lastly, the safety and efficacy of ADCs in combination with other drugs warrant further investigation. Additionally, the high cost and immature commercial production process of ADCs may impede their widespread adoption.

Taking into account these potential issues, we propose the following directions for ADCs development in urological cancers: On the one hand, it is vital to persistently advance the inherent technology of ADCs. This comprises the investigation of new-fangled payloads, the development of more reliable and stable linkers, and the refinement of payload connection methods. On the other hand, it’s also necessary for us to handle basic challenges, most notably the task of identifying suitable target sites: whether through large-scale sequencing for the direct identification of operational targets or by combining ADCs with other medications to transfigure originally unsuitable targets into appropriate ones. In terms of the application of ADCs in urological oncology, we must shape our research priorities based on the existing conditions of the three most prevalent types of urological tumors. For renal tumors, the quest for suitable ADC targets is of overriding importance. In the scenario of urothelial carcinoma, it is necessary to investigate strategies to mitigate the cost of ADC. As for prostate cancer, given the limited effectiveness of the majority of payloads, resolving sensitivity issues is a requirement. While these challenges are intricate and urgent, they also presage considerable market and developmental opportunities in the future landscape of ADCs.

This review focuses on the application of ADCs in urinary tumors, summarizing the applied and potential targets for various cancers, including renal, urothelial, and prostate cancers. Additionally, we catalogue the chemical agents coupled to different tumor types. As a result, this review offers guidance for the development and application of ADCs in urinary tumors. As a cutting-edge innovation borne out of precision medicine, the deployment of ADCs is still in its infancy within the field of common urological cancers. Given the scarcity of scientific research and clinical studies related to less prevalent urinary system cancers (such as adrenal and testicular cancers), and specific subtypes of urological malignancies (like upper urinary tract urothelial carcinoma), a comprehensive discussion on each is impossible. Moving forward, we aim to analyse long-term follow-up results of ADC treatments to further evaluate their safety, effectiveness, and potential strategies to address cost and production challenges.

## Method

A literature review was performed using the PubMed database to identify relevant studies on the clinical application, challenge, and prospect of ADCs in urinary tumors. The search was conducted from January 2011 to January 2023, and keywords such as antibody–drug conjugates, ADC, urothelial, bladder, prostate, kidney, urinary tumors, etc. were used to retrieve relevant articles. The purpose of this review is to collect and discuss the applications of antibody-drug conjugates (ADCs) in urological tumors, analysing the current status and challenges of ADCs in the treatment of urologic malignancies. The findings of this review will aid in enhancing our understanding of the use of ADCs in urological cancers. Using a database of search terms = A comprehensive search strategy was implemented to identify relevant studies for the literature review on the clinical application, challenge, and prospect of ADCs in urinary tumors.

To ensure the quality of the literature, papers presenting data in the form of letters, editorials, study protocols, case reports, short communications and articles not published in English were excluded. Colleagues examined the literature of all included papers for additional studies of interest. On this basis, 5 articles published before January 2011 and 9 article published after January 2023 were included.

Three independent researchers (two urology researchers, one clinical laboratory researcher) assessed each of these articles for quality and thematic suitability. If two researchers provided negative comments on a particular study, it was excluded from the final selection.

## Author contributions

KL: Conceptualization, Data curation, Formal Analysis, Methodology, Visualization, Writing – original draft, Writing – review & editing. GX: Conceptualization, Visualization, Writing – review & editing. XD: Data curation, Visualization, Writing – review & editing. YZ: Data curation, Resources, Visualization, Writing – review & editing. ZH: Conceptualization, Data curation, Software, Supervision, Writing – original draft, Writing – review & editing. ZJ: Conceptualization, Project administration, Supervision, Writing – review & editing.
